# Perceptions about Iranian-Kurds’ ethnic-inequality in health

**DOI:** 10.1186/s12914-017-0133-3

**Published:** 2017-09-12

**Authors:** Arezoo Yari, Saharnaz Nedjat, Mohsen Asadi-Lari, Reza Majdzadeh

**Affiliations:** 1 School of Public Health, Tehran University of Medical Sciences, Tehran, Iran; 2 Present address: Kurdistan Research Center for Social Determinants of Health (KRSDH), Kurdistan University of Medical Sciences, Sanandaj, Iran; 3 Knowledge Utilization Research Center (KURC), Tehran University of Medical Sciences, Tehran, Iran; 4 School of Public Health, Iran University of Medical Sciences, Tehran, Iran; 5 Oncopathology Research Center, Iran University of Medical Sciences, Tehran, Iran; 6 Community Based Participatory Research (CBPR) Center, Tehran University of Medical Sciences, Tehran, Iran

## Abstract

**Background:**

Evidence shows ethnic-inequality is a very effective variable in the Community and individual health associated outcomes. This study focused on gaining a deeper understanding of people’s perception on inequality of health in Iranian-Kurds and its determinants.

**Methods:**

The study was conducted in the three cities of Marivan, Sanandaj (capital of Kurdistan province in Iran) and Tehran (capital of the country). The study was conducted through 34 in-depth interviews and ten focus group discussions with health services users, academic graduates and health delivery service personnel.

**Results:**

Consensus on social, mental and physical health inequality did not exist within the study participants. However, there were concerns about differences in healthcare access and utilization. Several participants believed that access to health services and socio-cultural differences of Kurds affected the healthcare utilization.

**Conclusions:**

Since, people perceived ethnic-inequality in healthcare access and utilization, ethnicity must be considered as a mandatory stratifier in monitoring health status and a concern during planning health interventions. People’s awareness, resources management and allocation are factors requiring more consideration when choosing policy options.

## Background

Based on the different existing ethnicities, each country has its own specific ethnic classification criteria, which bring about difficulties when conducting studies related to this particular variable [1]. In recent years ethnicity has been under the spotlight as an aspect of social health and numerous articles have been published in this regard [2].

Some ethnic groups around the world have inferior health compared to other groups [3]. Ethnic-inequality in health is a situation where people from different ethnicities that share similar needs, receive unequal health care benefits and / or have higher morbidity and mortality rates which are not fair and can be avoided [4]. In both developed and developing countries, many minority groups receive lower income [1, 3], while also having inappropriate working conditions and live in environmentally and economically poorer areas with high dense populations [5]. Ethnic discrimination is a one of the causes for stress in these people’s lives which in turn also affects their health. This problem has been given much more attention during recent years [6, 7].

With a population of close to 80 billion, Iran has a vast ethnic variety [8]. Social aspects such as educational needs [9–11], marital patterns [11, 12], and gender inequality [13, 14] in different ethnicities have been given more attention in recent years.

**Fig. 1:**
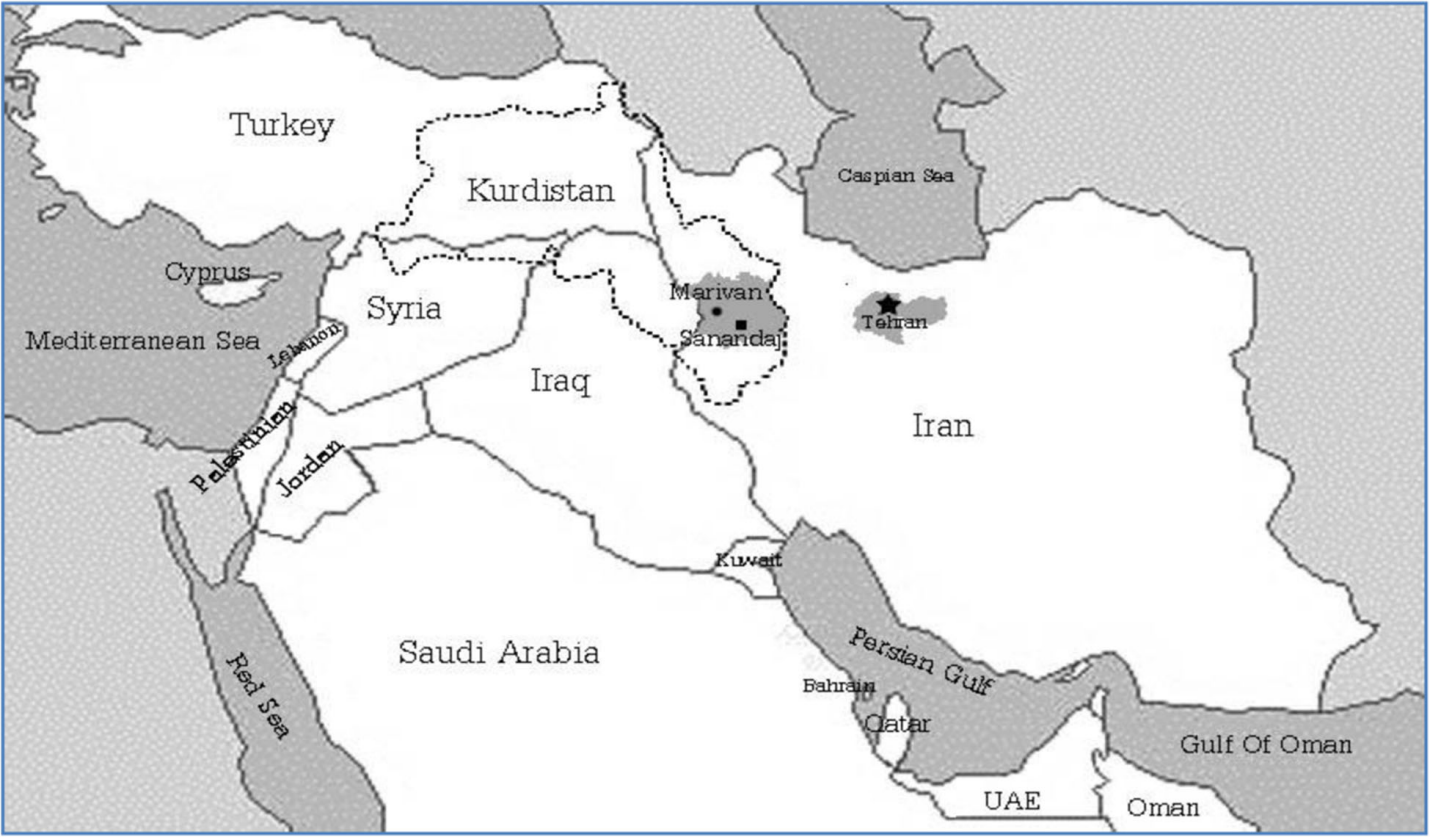
The habitat of the Kurds is represented by the dotted line shared between the borders of Iran, Iraq, Turkey and Syria. The shaded regions represent Marivan, Sanandaj and Tehran, where the research took place

Iran’s Kurdistan province is in the west and shares borders with Iraq. Figure 1 shows the Kurds’ habitats that are mostly living in the 4 countries of Iran, Iraq, Turkey and Syria. Kurdistan of Iran is of the less developed among the provinces with the lowest life expectancy rates [15] and human development index after Sistan and Baluchistan in south east [16]. The ethnic Kurd in Iran has almost two main sub-ethnic groups namely Sourani and Ourami.

Based on reviewing available studies, it can be concluded that the social aspects of ethnicities in Iran have been given more attention than health. Considering mass migration and changes in ethnic groups’ interactions in the modern era, the question is that do ethnicities still affect the health of the population? On the other hand, the perception of inequality is of significant importance regardless of the actual differences (whether they exist or not). Thus, knowing how people perceive ethnic inequality and its effect on their health is crucial.

Considering the ethnic diversity in Iran and the significant importance of the Kurd ethnicity, this study was conducted to understand the people’s perspectives about Iranian-Kurds’ ethnic-inequality in health and examines its determinants.

## Methods

This study was conducted in the 3 cities of Marivan, Sanandaj and Tehran during 2011–12. These cities have different variety of ethnic compositions and subsequently varied sensitivity to ethnicity. Marivan is in the Iran’s Kudistan located on the Iran-Iraq border. This city population consists of Sourani and Ourami, equally. So, the issue of difference between the two sub-ethnic groups has importance in this city. The majority of Sanandaj’s (the capital of Kurdistan province) population is Sourani. Here, the differentiation is between Kurds and non-Kurds. Tehran, the capital of the country, has drawn many Iranian ethnicities to itself and caused them to intermingle. The later city was chosen because it is where Kurds, as an ethnic minority group, live among a population with diverse ethnic mixes. In summary, in Marivan sub-ethnicities of one ethnic group, in Sanandaj Kurd ethnicity as a majority group and in Tehran Kurd ethnicity as a minority group were investigated.

This qualitative study was conducted using in-depth interviews and Focus Group Discussions (FGDs): 17 in-depth interviews and 5 FGDs in Marivan; 7 in-depth interviews and 2 FGDs in Sanandaj; 10 in-depth interviews and 3 FGDs in Tehran. In total, 34 in-depth interviews and 10 FGDs were conducted that are shown in Table 1. Purposeful sampling with maximum variation was applied to choose participants continuing the point of data saturation. The interviews were conducted in 3 separate groups of health delivery service personnel (HDSP), academic graduates (AG) and finally, healthcare services users (HSUs).

**Table 1:** Number of sessions with the study subjects according to the city and study group

Study group								
City	Health Services Users (HSUs)		Academic graduates (AGs)		Health Delivery Service Personnel (HDSP)		Total	
	FGD^a^	In-depth interview	FGD	In-depth interview	FGD	In-depth interview	FGD	In-depth interview
Marivan	1	8	0	4	4	5	5	17
Sanandaj	1	3	0	1	1	3	2	7
Tehran	1	3	0	3	2	4	3	10
Total	9	11	0	8	4	7	10	34

^a^Focus Group Discussion

The HDSP were chosen from the district health centers and one urban healthcare center in every city studied. In the formation of every FGDs, it was noted that all of the participants verbally confirmed their consent. An effort was made in all the three groups to interview both men and women of different socio-economic classes.

In case the number of interviewees reached the required minimum for a focus group discussion and provided they gave their consent to participate, the group discussion was conducted.

The interview was conducted using a questionnaire guide designed by an expert in the field of the given research with the aid of two qualitative study researchers. Interviewers were the researcher that has enough knowledge about the implementation of the study. Discussions’ guide for in-depth interviews and FGDs were developed according to the target groups (AGs, HSUs and HDSP) and the status of each city including language. It was pretested beofer the field work. The following issues were discussed and questioned from the interviewees: to what extent study subjects consider ethnicity in their identity; the effects of ethnicity on health; health inequality among various ethnic groups and the reasons behind it. The interviews were audio-recorded and transcribed verbatim.

Thematic analysis was used to analyze the data using the ‘Opencode’ software. Relevant codes and categories were extracted. The context of each interview was first considered as a whole, and then the general or fundamental meaning of the context was explained in one or more clauses. Primary codes and categories were designed and after numerous analyses, those that repeated were merged. Furthermore, to verify the study’s, two individuals coded the interviews. Finally, the codes were modified, merged and or removed by two other individuals.

## Results

An introductory question was asked in the FGDs: “how would you introduce yourself?”. The purpose of this question was to discover the significance of ethnicity in persons’ identity. Among the participants from AGs and HSUs in all three cities, seldom someone introduces her/him-self through their ethnicity. Only one person in Marivan addressed herself as a Kurd among ten interviews held with the HDSP, while most others spoke about their family, social matters and employment.

Since the main objectives of the study were recognizing the perception of people regarding health inequalities in Kurds and its determinant factors, the results section is accordingly divided to two parts of the participants’ views on health inequality and then the determinant factors. While presenting the study’s results, in addition to the explanations, certain quotes given by the interviewees along with the geographical location of study and group have been included in italic fonts.

### Health inequality

#### Physical health

None of the participants spoke about physical health, i.e. morbidity and mortality rates, inequality. Some mentioned the differences Kurds have in their lifestyles. A matter that was repeated during the interviews was the difference in physical activities among various ethnic groups. Moreover, most Souranis both in Marivan and Sanandaj commented on the Ouramis’ better health, and relate it to their ‘healthy lifestyle’.

Sanandaj - AG: *Being a Kurd does not affect one’s physical health whatsoever.*

#### Mental (psychological) health

Participants noted mental health inequality among ethnic and sub-ethnic groups, and the Kurdistan population’s worse mental health was underlined. However, some thought the opposite. In Marivan and Sanandaj most interviewees believed Ouramis enjoyed better mental health.

Marivan - AG: *No doubt our mental health (Kurds) is worse. We’ve probably seen more war, which is quite influential.*

Sanandaj- HSU: *Kurd’s women are aggressive; when someone is harsh their mental health is affected.*

Marivan - HDSP: *The Ouramis are happier and work harder, while the Souranis are more relax and easygoing.*

#### Social health

Participants did not have consensus on social health inequality. Some considered Kurds more violent than other ethnic groups, and perceived that it shows lack of social support. Others believed they are capable of rejoicing even after experiencing terrible deficiencies and miseries. One participant even went as far as completely denying the Kurds’ violence. Other participants thought other ethnicities in Iran, e.g. Gilaks and Lors, are even more violent than Kurds.

### Determinants of inequality

#### Access to health services (AHS)

Among reasons responsible for health inequality, the unequal distribution of facilities and healthcare were mentioned, repeatedly.

Marivan - HDSP: *An example is the differences in health facilities between two cities in two provinces. They are separated just by a single mountain. One city (advantaged) lies in the Kermanshah province while the other (disadvantaged) rests in the province of Kurdistan.*

Sanandaj- HSU: *Our health facilities are terrible. We even have to provide Vitamin A drops for ourselves from outside the facility.*

Contrary, some of the participants from Marivan believed they had no issues regarding healthcare services and that their situation was similar to other regions of Iran. Most interviewees believed Kurdistan’s primary health care facilities are equal to those of other provinces. The main differences are in the curative facilities especially the high-technology-equipped ones.

Marivan - AG: *We don’t even have a private hospital in our city!*

Others believed the difference in AHS is related to geographic situation, such as being in remote mountainous regions or border areas, and is not related to ethnic discrimination.

Tehran - AG: *Most provinces close to the border are seldom looked upon; they don’t have health facilities. The farther they are, the less their facilities become.*

Tehran - HDSP: *This has nothing to do with ethnicity, yet has everything to do with the drawbacks of certain regions. Some of them have been left behind.*

These people believed that the differences here are not just due to the ethnicity and other factors can affect different distribution of health. They said that being situated on the border has influence on disease transmission and its effects on the health of Kurds and also other ethnicities.

Sanandaj - HDSP: *The fact of being close to or on the borderline can increase the transmission of certain diseases. This is what can affect health; eventually there will be epidemic and communicable diseases over there and they could be transmitted to other side.*

In addition, these participants pointed out other factors of the effects of being situated close to, or on the borderline such as the formation of illegal jobs especially smuggling, the region fragility towards natural disasters, the long term impact of war and etc.

Sanandaj - HSU: *Like Khuzestan on the border with Iraq, the war, its aftermath - ...- cultural influences - increase in addiction in Sistan and Baluchistan.*

However, positive effects such as trade, economic prosperity as a result of connections with the other side of the border were also mentioned. Those who believed on ethnic-inequality in the AHS perceived the effect of three factors on this inequality:

1- Politics and governance: A number of interviewees, particularly those from Sanandaj noted the possible relationship between health problems and political issues. This may prevent huge investments and affect people’s AHS.

Sanandaj - AG: *the province of Kurdistan has been wrongly introduced as a region where many political problems exist, and they’ve accentuated these issues.- ….- The fact that we don’t have a very large hospital with excellent facilities is because they say this place is unsafe, and that has had its (negative) effect.*

2- Economic status: The economic inequality of various ethnic groups and even sub-ethnic groups were mentioned in the interviews as one of the reasons behind health inequality.

Sanandaj- HSU: *Someone with a better economic status has better mood and is happier.*

Tehran - HDSP: *Eventually, economics affects the situation. For instance, oil affects the economic status of those living in the South; whereas those living in the East can’t earn that much from crops.*

3- Distribution of Human resources: Some blamed the public administrators for the improper distribution of human resources, not employing local manpower and their negligence. In their opinion, mismanagement was the main reason for this issue.

Tehran - HDSP: *The state should facilitate the deprived regions as doctors prefer to practice in those areas. Government policies are very important; showing how much it believes in the development of social equity and widespread facilitation.*

However, some others believed that it’s not the public administrators’ fault.

#### Socio-cultural characteristics

The socio-cultural differences between the two Sourani and Ourami sub-ethnic groups were pointed out within Kurdistan. This determinant has the following categories:

1- Beliefs and traditions: One problem affecting people’s healthcare utilization (HCU) is their culture of acceptance and the people’s negligence. However, they said that the differences can be due to the service delivery method and difficulties to get the service when needed.

2- Hygien and nutritional status: From the point of views of the participants’ in Marivan, one factor affecting health in ethnic and sub-ethnic groups is hygiene and nutritional status. Interestingly Ouramis believed they are living more hygienic. No one in Sanandaj mentioned this and this issue was discussed only in one of the interviews with HDSPs in Tehran.

Marivan - AG: *When you go to an Ourami village there are no bad smells, but when you go to a Sourani village, there are animal droppings and….. they make it a non-hygienic environment.*

3- Social networks: Some people noted the weakening social networks that can affect the health of different ethnicities through social and psychological supports, exchangeability of information and behaviors.

Marivan- HSU: *If one person is an Ourami, and I also belong to Ouramis’, they will take me more seriously than a person who is Sourani.*

## Conclusions

The present study’s findings show, based on perception of the study subjects, there is no concern on inequality of physical health, different points of view exist regarding mental and social health, but there is a common belief about AHS inequalities.

The first point is that practically no one mentioned her / his ethnicity during the briefings. This could be because of the lack of ethnic differentiation. The ethnic-inequality in lifestyle, which is a risk factor for health in different dimensions, was clearly pointed out. In other parts of the results, inequalities is in due to various social and cultural characteristics; the effects of tradition, hygiene and nutrition were mentioned which can be the source inequality in life style as well. Other studies on health inequalities on cardiovascular diseases, diabetes, asthma and cancer found social determinants as the most prominent reason behind these differences [17–19].

Although there was no general consensus on mental and social health inequalities, but the fact remains that they were given more attention. But some believe that the state of mental health for the Kurds to be unsatisfying. One study shows the rate of violence against women to be significant in Marivan [20]. According to a National Health Survey, the prevalence of mental illnesses in individuals older than 15 years of age in Kurdistan was 21.8%, ranking 13th among 30 provinces [15].

The present study shows that what people perceived is inequality in AHS. This inequality is obviously associated with health outcomes and affects the ethnic groups’ sense of prejudice and dissatisfaction of healthcare services [21, 22]. This perception of inequality can be classified into 3 groups: A) Problems and issues that cause an actual perception of deprivation backed by logical aspects. For example, the participants pointed out the AHS inequalities between Kurdistan and neighboring provinces. B) Although certain inequalities exist, some of them are not due to the public sectors’ mismanagement. For example, the absence of a private hospital is associated with the lack of private sector interest. C) A number of demands that are not realistic and may be considered false; for example, some expect high-tech medical facilities to be set up in small cities, which is not rational considering the population size and subsequently the number of patients. After the World Health Organization’s 2008 report on primary health care, equity in affordable HCU was recognized as a significant challenge in the current health systems, for which the ‘universal health coverage’ targets were proposed [23]. The most prominent step in Iran’s health system was the ‘family physician program’ and later on the Health Transformation Plan. This program was yet another solution to the aforementioned problem, designed to increase access to quality health services and backed by a desirable insurance policy [24]. Nevertheless, the rational issues put forth by the people must be taken into careful consideration by the authorities. In order to eliminate the perception of prejudice, people must be informed about the justifiable issues (such as the absence of a private hospital or other irrational demands).

According to the present study’s findings, another reason responsible for health inequalities was the human resources’ lack of interest, physicians in particular, to be in the deprived regions of Iran. Kenneth Wells says, considering the inequalities in health accessibility and uncertain need and quality of mental healthcare, we lay emphasize on the importance of general education, intervention on physicians performances and other HDSP in order to improve the quality and situation of ethnic minorities’ healthcare [8]. By intervening in the country’s healthcare system’s operation in delivering equal services to regions with specific strategic conditions, and paying attention to ethnic differences, the feeling of inequality can be reduced.

In this study, people considered a social network as a socio-cultural characteristic that led to the bondage or support of similar groups. Nazeroo and Karlson have also shown that the formation of social networks in various ethnic groups may lead to interpersonal or organized prejudice, affecting personal health [5].

Another finding in our research was the health inequality existent among various ethnic groups, which is mainly because of the ‘different’ acceptance of new health issues. Lack of information, low level of education, cultural incompatibility between ethnic groups and HDSP and socio-cultural barriers and differences are why new issues are accepted and comprehended differently. Research has shown the relationship between HDSP and patients to be directly related to the patients’ satisfaction, obedience and side effects of their health [25, 26]. As the studies show, when a country has a vast variety of ethnicities and cultures, the HDSP face a range of patients from different ethnic groups, where each have a unique understanding of the health system [19, 27–29].

In conclusion, this study provides evidence to policymakers, especially those who have concern over UHC, to informed design interventions on AHS and HCU by considering ethnic and sub-ethnic perceptions. The people’s lack of awareness, their undesirable culture regarding the acceptance of health services, poor AHS, the service delivery system’s problems and the unequal distribution of human resources were stated as factors affecting HCU. The perceptions behind inequality and inappropriate AHS were the unsuitable financial status of the province of Kurdistan and also the reputation of political disputes. It seems that ethnic variables should be considered as an essential element of the Iran’s health information system for monitoring health inequality to be used in further interventions.

## Abbreviations

AG: Academic Graduates
AHS: Access to Health Services
FGDs: Focus Group Discussions
HCU: Healthcare Utilization
HDSP: Health Delivery Service Personnel
HSU: Health Services User
IRB: Institutional Review Board
